# Erythromycin: an alternative for the management of oral mucositis?

**DOI:** 10.4317/medoral.25439

**Published:** 2022-06-19

**Authors:** Dieni Silveira Teixeira, Gabriel Campos Louzeiro, Maria Antonia Zancanaro de Figueiredo, Karen Cherubini, Fernanda Gonçalves Salum

**Affiliations:** 1 PhD. School of Health and Life Sciences, Pontifical Catholic University of Rio Grande do Sul-PUCRS, Porto Alegre, Brazil; 2 PhD. Oral Medicine Division, Pontifical Catholic University of Rio Grande do Sul-PUCRS, Porto Alegre, RS, Brazil

## Abstract

**Background:**

Oral mucositis (OM) is an important acute adverse effect of anticancer therapy. This condition presents high morbidity and may lead to the suspension of anticancer therapy.

**Material and Methods:**

We reviewed the literature on the pathobiology of OM and the properties of erythromycin (EM), to consider the possibility of its use for the prevention and treatment of OM. We searched the PubMed, Scopus and Web of Science databases and selected complete articles published in English or Spanish that met the inclusion criteria. The search terms “erythromycin”, “inflammation”, “immunomodulation” and “oral mucositis” were used.

**Results:**

The control of free radicals, transcription factors and pro-inflammatory cytokines has been considered as the key to the management of OM. EM has the ability to modulate oxidative stress, acts on the transcriptional system and inhibits the production of several cytokines that have been directly implicated in OM pathobiology.

**Conclusions:**

The present review suggests that EM could be effective in the treatment of OM. Experimental studies investigating the use of EM in OM should be encouraged.

** Key words:**Oral mucositis, erythromycin, inflammation, immunomodulation, oxidative stress.

## Introduction

Oral mucositis (OM) is an important acute adverse effect of anticancer therapy and can be considered the most serious non-hematological complication of oncologic treatment. Clinically, it is characterized by the presence of erythema, erosive lesions and painful ulcers. It may be accompanied by dysphagia, taste changes, weight loss and opportunistic infections (1). The incidence and severity of lesions depends on the therapeutic regimen employed. It occurs in approximately 20 to 40% of patients receiving conventional chemotherapy (CT), 60 to 85% of the ones undergoing high-dose CT as conditioning for hematopoietic stem cell transplantation and 80 to 100% of patients receiving head and neck radiotherapy (2). OM presents high morbidity and may lead to the suspension of anticancer therapy, negatively interfering with the patient's prognosis.

The pathogenesis of OM is a dynamic process, classically divided into five stages: initiation, upregulation, signal amplification, ulceration and healing. Ionizing radiation and CT induce direct DNA damage, generation of reactive oxygen species (ROS), and death of basal epithelial cells. Afterwards, there is activation of the innate immune response and nuclear transcription factors and the production of inflammatory cytokines. Such events lead to signal amplification and consequent ulceration. At the end of antineoplastic treatment, cell migration and proliferation occur, culminating with healing (2-3). Interventions to prevent and reduce the incidence of these lesions are necessary; however, there is no gold standard substance for the treatment of OM.

Erythromycin is a 14-member macrolide originally discovered in the 1950s. It has broad-spectrum antimicrobial action, inhibiting bacterial protein synthesis. Studies have shown that erythromycin has immunomodulatory and anti-inflammatory activity independent of its antimicrobial action (4-6). This substance can suppress the production of inflammatory cytokines and activation of nuclear transcription factors and can promote the apoptosis of inflammatory cells (7). Furthermore, it can inhibit bacterial biofilm formation even in acute wounds (8). It was reported that the use of topical erythromycin in the oral cavity was able to reduce the inflammatory process of traumatic ulcers, favoring tissue repair (9).

Thus, due to its properties, mechanism of action and favorable results in the treatment of inflammatory disorders, we consider the use of erythromycin in the management of OM. Therefore, the objective of this review was to evaluate the therapeutic viability of erythromycin in the prevention and treatment of OM on the basis of the available scientific literature.

## Material and Methods

Accordingly, we carried out a literature review in the PubMed, Scopus and Web of Science databases. We used the terms "erythromycin”, "inflammation", "immunomodulation" and "oral mucositis" to search for full articles published in English or Spanish. There were no restrictions regarding the year of publication and the studies retrieved were published before May 2021. All types of articles (pre-clinical studies, clinical trials, review articles) were included. Additional papers were obtained from the reference lists. We selected studies investigating mechanisms of action, indications and possible uses of erythromycin. Publications regarding the pathobiology, clinical aspects and forms of treatment of OM were also included.

## Results

A total of 583 papers were found in the databases. After exclusion of duplicated papers, the title and abstracts of the papers were reviewed. The papers which fulfill the inclusion criteria of the pre-sent study were included, as well as the references within them.

- Oral mucositis

OM is classified as an epithelial and subepithelial injury secondary to antineoplastic therapy (3,10). Among patients receiving CT or myelosuppressive CT, the first signs of OM (erythema and atrophy) generally appear about three or four days after the beginning of therapy and the formation of ulcerated lesions follows. Between the first and second weeks, the cytotoxic effect gradually becomes more severe, showing greater intensity of OM. In most cases, the ulcers spontaneously heal at the end of the third week (1-2).

The lesions are typically very painful and may require the use of opioid analgesics (10). Consequently, reduction in the quality of life of these patients due to dysphagia with solid and liquid foods, lack of speech muscle coordination and pain or discomfort, while swallowing has been reported (1). Furthermore, ulcers are a potential source of septic complications because of the loss of protective epithelial and basement membrane barriers. The literature presents some factors related to patients that can contribute to the onset of OM, such as age older than 65 years, poor oral hygiene, periodontitis, salivary gland dysfunction, below average nutritional status, chronic diseases (e.g., diabetes) and smoking. Other risk factors include CT dose and protocol, concomitant head and neck radiotherapy (1,11).

Over the years, several grading scales have been proposed. Table 1 shows the comparison of different OM scoring scales (12-14). The use of these instruments allows the assessment of aspects related to the signs, symptoms and morbidity associated with injuries. In addition, the standardization of clinical analysis is important to assess the effectiveness of a specific treatment. The National Cancer Institute (NCI) and World Health Organization (WHO) criteria are widely used for simplicity and ease of application because they combine objective and functional aspects (15). The Radiation Therapy Oncology Group (RTOG) scale is used to evaluate objective and subjective criteria associated with radiotherapy-induced OM (14).

The management of the most severe cases of OM (grades 3 and 4) is a challenge for physician, since severe OM limits therapeutic doses of CT and radiotherapy and changes clinical protocols. Another important factor is the considerable economic impact, due to costs associated with the treatment of signs and symptoms and the need for hospitalization. In this perspective, several substances have been employed in an attempt to reduce the incidence and severity of lesions, as well as to favor the repair process.

**Table 1:**
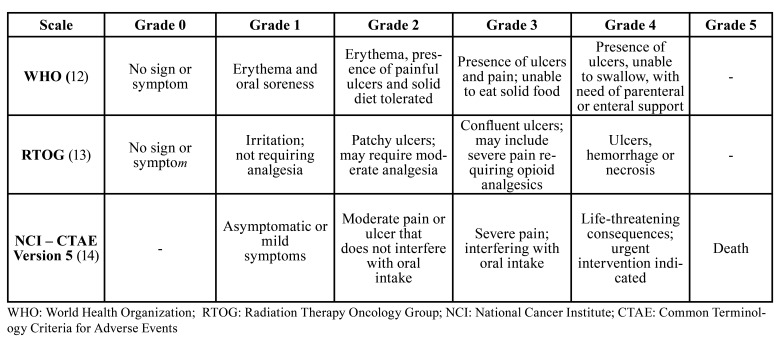
Oral mucositis grading scales.

The use of anti-inflammatory substances, systemic antimicrobials, topical anesthetics, laser therapy, cryotherapy and growth factors has been highlighted by several studies (10,16-17). The only agent approved by the Food and Drug Association (USA) is palifermin (recombinant human keratinocyte growth factor), but its use is limited to patients with hematologic malignancies who received myelotoxic therapy, besides being limited due to the considerable associated cost (16).

Pathobiology of OM

Initially, it was believed that the damage caused by antineoplastic treatment would be limited to the oral and gastrointestinal epithelium. Radiotherapy and most CT agents do not act selectively and kill both malignant and normal cells, especially fast-growing cells (2).

**Figure 1:**
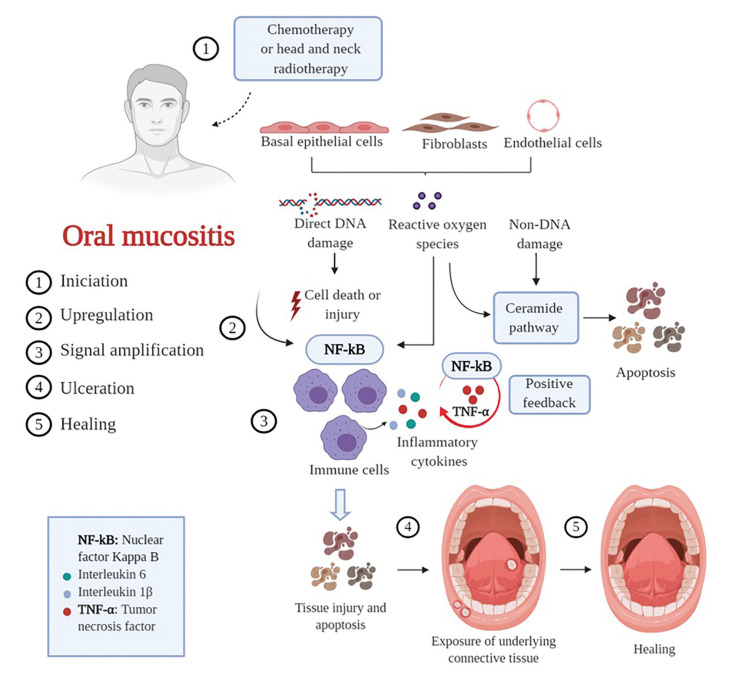
Model of pathobiology of oral mucositis. Created with BioRender.com.

In 2004, Sonis (3) proposed the five-stage model indicating that the initial damage occurs in basal layer and submucosal cells (Fig. 1). Direct damage consists of DNA strand breaks and indirect damage of the generation of free radicals, which contribute to the onset of oxidative stress. In the next stage, these biological events induce the activation of several transcriptional pathways, such as nuclear factor kappa B (NF-κB). This factor is responsible for the upregulation of more than 200 genes involved in the production of pro-inflammatory cytokines and enzymes that result in DNA damage, cell injury and cell death.

In the signal amplification phase, inflammatory cytokines have the ability to upregulate transcription factors (positive feedback). This effect is observed mainly between tumor necrosis factor-α (TNF-α) and NF-κB, resulting in direct tissue damage and major structural changes in the submucosa. The epithelium then breaks down leading to the ulceration phase, in which there is the formation of deep, broad and painful lesions, covered by a pseudomembrane composed of cell remnants and fibrin, a clinical feature that favors microbial colonization. Gram-positive and Gram-negative bacteria may be able to invade tissues, inducing bacteremia. In the case of neutropenic patients, secondary bacterial colonization creates a significant risk of sepsis. In addition, products from the bacterial cell wall stimulate the production of inflammatory cytokines and release of metalloproteinases, which contribute to increased tissue injury.

After antineoplastic therapy has been completed, the tissue is gradually recovered with activation of enzymes that enhance angiogenesis. In the healing phase, the extracellular matrix provides signals to the epithelium influencing cell migration, proliferation and differentiation, promoting the reconstruction of the submucosa and the epithelial barrier.

- Erythromycin

Erythromycin (EM) is part of the macrolide antibiotic group, having a lactone ring with 14 carbon atoms. Isolated from the culture of Streptomyces erythreus, it was the first macrolide introduced in clinical practice. It has good tissue penetration and antimicrobial activity, especially against Gram-positive cocci and to lesser extent Gram-negative bacteria (18). Erythromycin has been used for treating various respiratory infections, prophylaxis of neonatal conjunctivitis, skin infections, rheumatic fever prophylaxis, syphilis, and pelvic inflammatory disease. Its mechanism of action is based on the inhibition of protein biosynthesis by binding to the 50S ribosomal subunit of susceptible bacteria. This drug does not act on the 40S and 60S ribosomal subunits, which are specific to mammalian cells. The side effect of erythromycin includes nausea, abdominal pain, and diarrhea (19).

It has been reported in the literature that macrolides have the ability to modify the inflammatory response and regulate one or more functions of the immune system (20). The immunomodulatory activity of EM has been extensively investigated in respiratory disorders, characterized by exacerbated inflammatory response and deleterious effects on tissues. Table 2 presents pre-clinical studies showing the effects of EM on cell function, inflammatory cytokines and oxidative stress (7,21-31).

Cytokine responses and signaling pathways 

Cytokines and chemokines have been described as regulatory keys for inflammatory response (32). As shown in Table 2, EM has demonstrated the ability to suppress or inhibit the production of pro-inflammatory cytokines such as interleukin 1β (IL-1β), interleukin 6 (IL-6), interleukin 8 (IL-8) and TNF-α in response to specific stimuli. The production of these cytokines is carried out by a variety of cell types, including macrophages, eosinophils, neutrophils, lymphocytes and bronchial epithelial cells and are responsible for amplifying the immune response through positive feedback loops (7,29,32).

There seems to be a consensus among authors that such evidence would be related to EM ability to alter intracellular signaling, particularly through the inhibition of NF-κB activation and expression of activator protein 1 (AP-1). NF-κB is one of the most studied factors and responsible for the regulation of more than 200 genes; it has been associated with immune responses, inﬂammation and cancer (33). Ren *et al* (4) investigated the effect of EM on murine macrophage cells stimulated by bioparticles.

**Table 2:**
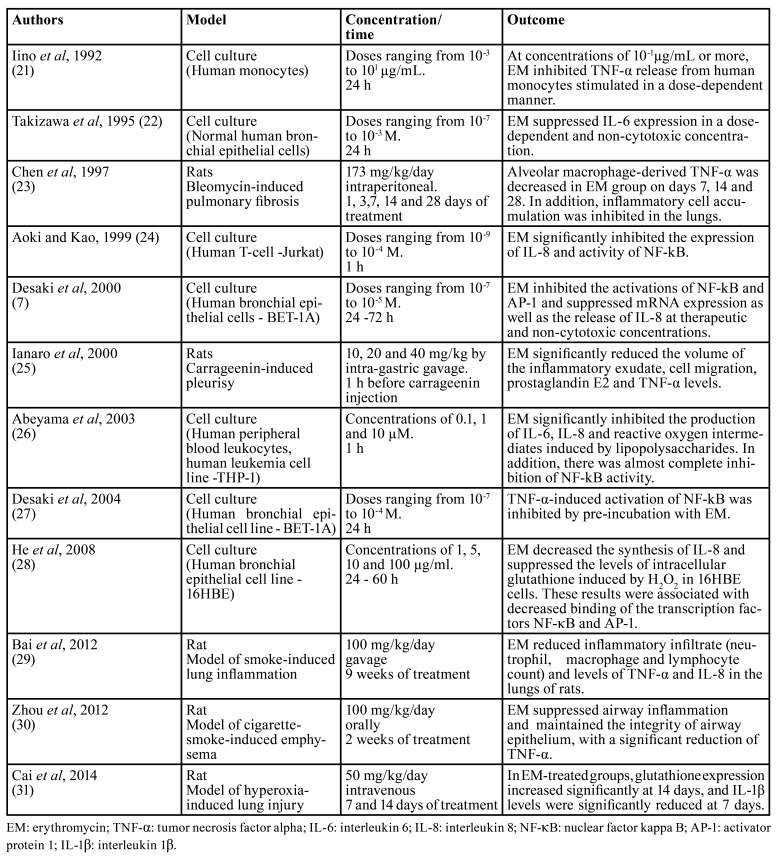
Pre-clinical studies involving immunomodulatory effects of erythromycin in airway diseases.

Pre-treatment with EM (5 pg/ml) significantly reduced NF-κB activity and the gene copy number of IL-1β and TNF-α. Similar results were observed by Uchimura *et al* (5) in an osteoarthritis model and Hirano *et al* (6) in autoimmune myocarditis in rats. The authors suggest that the effect of EM on signaling pathways and cytokine production may result in the modification of cell functions such chemotaxis, phagocytosis and oxidative burst, favoring the resolution of prolonged inflammation.

EM and oxidative stress 

ROS are unsTable molecules, products of normal cellular metabolism, and have an important role in cell signaling and homeostasis (34). Overproduction of these molecules results in oxidative stress, which can be induced by various factors, such as infection, inﬂammation, hypoxia and ionizing radiation (30). The antioxidant defense system has the function of inhibiting and reducing damage caused by the harmful action of free radical homeostasis (34).

Studies suggest that macrolide antibiotics have intrinsic antioxidant properties at the level of reducing the generation of ROS or increasing intracellular tolerance to ROS (18,26,28). Katayama *et al* (35) evaluated the neuroprotective effects of EM against cerebral ischemia in rats. Treatment with EM (50 mg/kg) suppressed post-ischemic lipid peroxidation products and oxidative DNA damage and reduced the inflammatory process. The authors suggested that an antioxidant effect was clearly mediated through the suppression of ROS.

The increase in intracellular tolerance to free radicals can be observed from the activity of the enzymatic antioxidant system. Superoxide dismutase and glutathione play an important role in the prevention of damage induced by ROS (28). Morikawa *et al* (36) assessed the effect of EM in patients with diffuse panbronchiolitis and observed an increase in superoxide dismutase activity. Similarly, Cai *et al* (31) observed an increase in glutathione activity in rats treated with EM in a model of hyperoxia. These findings were accompanied by suppression of inflammatory cytokines and attenuation of the clinical features.

## Discussion

OM is a very common, potentially severe side effect, caused by anticancer therapy. The cytotoxic effect on the oral mucosa, causes an imbalance in the redox state and stimulates the production of pro-inflammatory mediators responsible for tissue damage. We hypothesize that EM would present properties that could assist in the management of this condition, reducing the incidence and severity of the lesions. However, there are no studies in the literature, so far, related to the use of EM for OM.

In airway disease, the immunomodulatory properties of EM have been investigated for over 40 years (18). Signaling pathways, cytokine responses and oxidative stress were among the main research targets (20). In oral medicine, studies involving the anti-inflammatory properties of EM are scarce. Teixeira *et al* (9) investigated the topical effect of EM in traumatic ulcers. The use of EM reduced local inflammation, and this outcome was accompanied by clinical improvement of the lesions.

In this study, the review of the mechanisms of OM pathobiology highlighted the importance of oxidative stress for the onset and maintenance of lesions. Drugs that target the control of free radicals and the antioxidant system have shown good results (10,37). As explained, pre-clinical studies have demonstrated that EM has the ability to modulate the production of ROS, as well as increasing intracellular tolerance to free radicals (18,26,28). We believe that these characteristics could delay the appearance of lesions, as well as prevent the occurrence of the highest degrees of OM. In addition, EM can modulate the oxidative explosion of phagocytes, which could limit the tissue damage caused by macrophages during the ulcerative phase (38-39).

Another important point is the participation of ROS in the activation of transcription factors. In the upregulation and signal amplification stages, NF-κB activity is essential for the modulation of pro-inflammatory cytokines. Modulation of the expression of transcription factors has been postulated as one of the main pathways by which EM exerts its immunomodulatory activity. Wu *et al* (40) evaluated the activity of EM in TNF-α-stimulated T cells and observed the inhibition of NF-κB. Moreover, EM influenced the proliferation and apoptosis of these cells. These effects could prevent the progression of OM, as well as favor tissue repair.

When considering EM as an alternative to treat complications of cancer therapy, it is important to evaluate its interaction with tumor cells. It is speculated that drugs able to protect healthy tissues could also protect neoplastic cells. Hamoya *et al* (19) investigated the chemopreventive effect of EM on mouse intestinal tumors. The authors observed that treatment with EM was able to suppress the development of intestinal polyps and decreased the levels of IL-6 and cyclooxygenase-2. These results suggest that the anti-inflammatory and antioxidant properties of EM would not have a protective effect on cancer cells, and conversely, EM would have a preventive effect on carcinogenesis.

In view of the evidence presented in the literature, we believe it is very important to investigate EM in OM models to determine its true potential.

## Conclusions

Although the scientific evidence is based on pre-clinical and few clinical studies, the present review suggests that EM could be a therapeutic option in the management of OM. The immunomodulatory effects of EM are probably responsible for the beneficial effects of this drug in the treatment of various inflammatory disorders. Studies investigating the use of EM in OM should be encouraged.
